# Supplementary material for: Design and Fabrication of Nanofibrous Dura Mater with Antifibrosis and Neuroprotection Effects on SH-SY5Y Cells
Source: Polymers (Basel). 2022 May 5;14(9):1882. doi: 10.3390/polym14091882 (PMC9099771; doi:10.3390/polym14091882)
Supplement: Supplementary file 1 [file polymers-14-01882-s001.zip › polymers-1614649-supplementary.pdf]

Supplementary information

Design and Fabrication of Nanofibrous Dura Mater with Antifibrosis and Neuroprotection Effects on SH-SY5Y Cells

Zhiyuan Zhao [1,2,3,†], Tong Wu [2,3,†], Yu Cui [2,3], Rui Zhao [1,2,3], Qi Wan [2,3,*] and Rui Xu [1,3,*]

1 Department of Interventional Radiology, The Affiliated Hospital of Qingdao University, Jiangsu Road 16, Qingdao 266000, China; qduzzy@126.com (Z.Z.); zhrui97@163.com (R.Z.)

2 Institute of Neuroregeneration and Neurorehabilitation, Qingdao University, Qingdao 266071, China; twu@qdu.edu.cn (T.W.); cuiyu1216@126.com (Y.C.)

3 Qingdao Medical College, Qingdao University, Qingdao 266071, China

* Correspondence: qiwan1@hotmail.com (Q.W.); xray3236@126.com (R.X.)

† These authors contributed equally to this work.

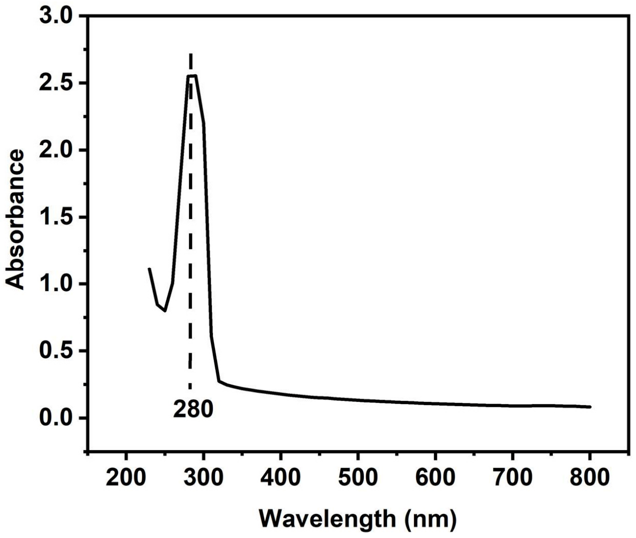

Figure S1. The ultraviolet full-wavelength scanning spectrum of TMP.

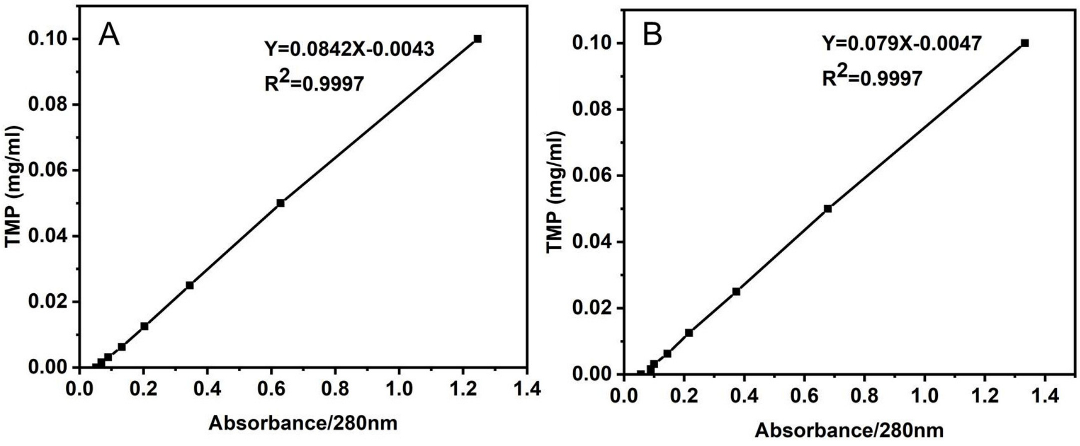

Figure S2: (A) TMP standard curve made with DCM as solvent for calculation of EE. (B) TMP standard curve made with PBS as the solvent for calculation of TMP release amount.

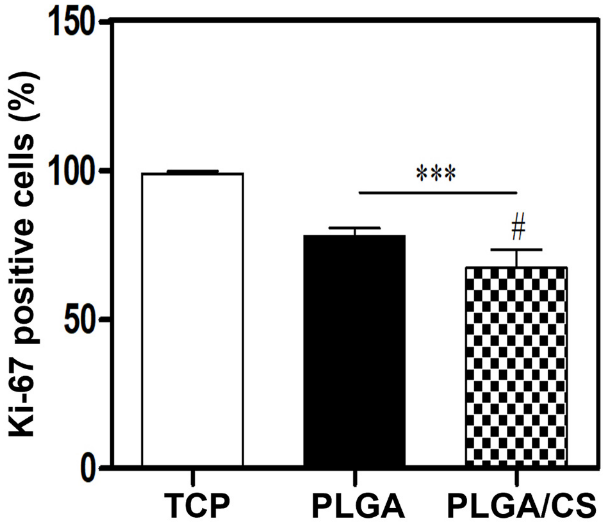

Figure S3: Percentage of Ki-67 positive cells as in figure 4. *** $p < 0.001$ as compared with TCP. $^{\#}$ $p < 0.05$ as compared with PLGA NDM.

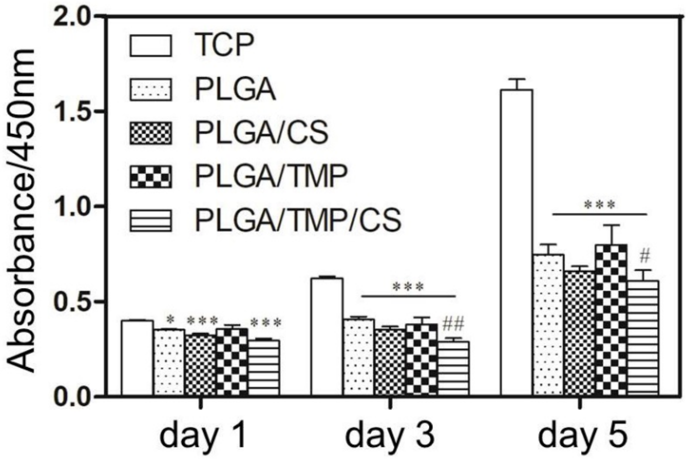

Figure S4. Cell viability of fibroblasts seeded on seeded on glass slides as the control group (TCP), on PLGA NDM, PLGA/CS NDM, PLGA/TMP NDM and PLGA/TMP/CS NDM after culture of 1, 3 and 5 days. * $p < 0.05$ and *** $p < 0.001$ as compared with TCP. # $p < 0.05$ and ## $p < 0.01$ as compared with PLGA/TMP NDM. All data are representative of or combined from at least three independent experiments.

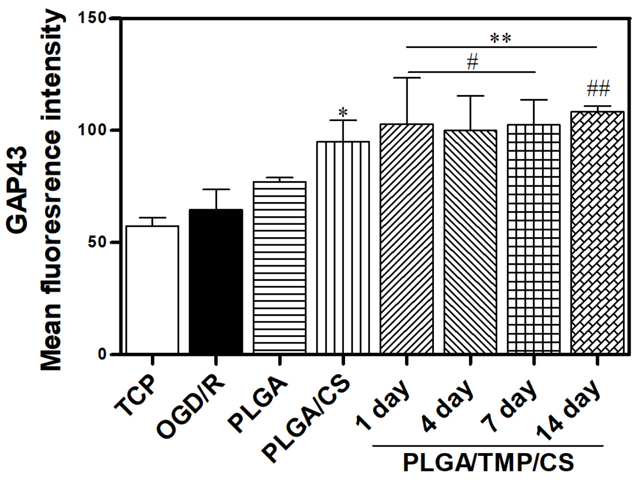

Figure S5: Expression of GAP43 of OGD/R-treated-SH-SY5Y cells as in figure 6. * $p < 0.05$ and ** $p < 0.01$ as compared with TCP. # $p < 0.05$ and ## $p < 0.01$ as compared with OGD/R.
